# Characterization of fine particulate matter from indoor cooking with solid biomass fuels

**DOI:** 10.1111/ina.13143

**Published:** 2022-11-18

**Authors:** Axel Eriksson, Asmamaw Abera, Ebba Malmqvist, Christina Isaxon

**Affiliations:** ^1^ Division of Ergonomics and Aerosol Technology Lund University Lund Sweden; ^2^ Ethiopia Institute of Water Resources Addis Ababa University Addis Ababa Ethiopia; ^3^ Division of Occupational and Environmental Medicine Lund University Lund Sweden

**Keywords:** charcoal, indoor exposure, PM_2.5_, Sub‐Saharan Africa, wood

## Abstract

Household burning of solid biomass fuels emits pollution particles that are a huge health risk factor, especially in low‐income countries (LICs) such as those in Sub‐Saharan Africa. In epidemiological studies, indoor exposure is often more challenging to assess than outdoor exposure. Laboratory studies of solid biomass fuels, performed under real‐life conditions, are an important path toward improved exposure assessments. Using on‐ and offline measurement techniques, particulate matter (PM) from the most commonly used solid biomass fuels (charcoal, wood, dung, and crops residue) was characterized in laboratory settings using a way of burning the fuels and an air exchange rate that is representative of real‐world settings in low‐income countries. All the fuels generated emissions that resulted in concentrations which by far exceed both the annual and the 24‐hour‐average WHO guidelines for healthy air. Fuels with lower energy density, such as dung, emitted orders of magnitude more than, for example, charcoal. The vast majority of the emitted particles were smaller than 300 nm, indicating high deposition in the alveoli tract. The chemical composition of the indoor pollution changes over time, with organic particle emissions often peaking early in the stove operation. The chemical composition of the emitted PM is different for different biomass fuels, which is important to consider both in toxicological studies and in source apportionment efforts. For example, dung and wood yield higher organic aerosol emissions, and for dung, nitrogen content in the organic PM fraction is higher than for the other fuels. We show that aerosol mass spectrometry can be used to differentiate stove‐related emissions from fuel, accelerant, and incense. We argue that further emission studies, targeting, for example, vehicles relevant for LICs and trash burning, coupled with field observations of chemical composition, would advance our understanding of air pollution in LIC. We believe this to be a necessary step for improved air quality policy.

**Practical Implications**

This study, looking at emission levels and physicochemical properties of airborne particles smaller than 2.5 μm, was conducted at an air exchange rate of 15 h^−1^, which is the average air exchange rate that was found in our recent field studies (26 homes) in Ethiopia. That means that the particle concentrations in the measurement volume, and the aerosol dynamics taking place, are relevant for real‐world indoor air in a Sub‐Sahara African county like Ethiopia. The results from this study can be used both for indoor exposure assessment in epidemiological studies, for source apportionment, and as input for more detailed toxicological studies of solid biomass emissions.

## 1 INTRODUCTION

It is well established that household burning of solid biomass fuels, such as charcoal, wood, crops residue, and dung, emits high levels of pollutants that are detrimental to human health. The World Health Organization (WHO) estimates that almost 3 billion people globally are dependent on solid biomass fuels for cooking and heating.^1^ According to the International Energy Agency (IEA),^2^ 900 million people in Sub‐Saharan Africa relied on solid biomass fuels in 2018. In Ethiopia, that percentage is >90% according to WHO.^3^ Africa is, hence, burdened by air pollution emissions from biomass burning,^4^ and due to the rapid increase in population, solid biomass fuel emissions are expected to escalate even more. This is mainly due to that the frequent challenges with erratic electric power^5^ are forecasted to remain; the IEA^2^ estimates that by 2030, over 600 million people in Africa will still not have access to reliable electric power.

Many low‐income countries (LICs), where solid biomass fuel is used for cooking, still have a long way to go toward compliance with the “strong recommendation” from WHO that indoor PM_2.5_ (particulate matter with an aerodynamic diameter <2.5 μm) must be reduced to values close to 10 μg/m^3^ ^6^ to avert most of the adverse effect on health caused by household air pollution, and an even longer way toward compliance with the ambient PM_2.5_ guideline of 5 μg/m^3^ as annual average.^7^ Taking Ethiopia as example, in Addis Ababa the average particulate matter (PM) levels during coffee making using charcoal found in one study^8^ were 905 μg/m^3^ in the personal breathing zone and 845 μg/m^3^ in the room's background air. In another Addis Ababa study,^9^ indoor PM_2.5_ measured in 59 homes in slum neighborhoods showed a 24‐h average of 818 μg/m^3^. A quadruple increase in PM_2.5_ was shown when animal dung rather than other biomass fuels was used. Additionally, a third study^10^ found that women are exposed to PM_2.5_ levels 7 times higher than what men are in Uganda and Ethiopia.

From a health perspective, exposure to household air pollution is globally the single most important environmental risk factor.^11^ Most particulate matter emitted from solid biomass fuels is smaller than 2.5 μm,^12^ enabling a predominant deposition in the deep lung. In 2012, household air pollution was responsible for 4.3 million deaths globally and nearly 5% of the global disease burden.^13^ Of these deaths, almost 600 000 occurred in Africa.^13^ Huang et al^14^ report that globally, PM_2.5_ and ozone from solid biofuel stoves emissions in ambient air (i.e., excluding the majority of exposure which occurs indoors near the stove) contributed to 382 000 premature deaths in 2010, corresponding to 8.1 million years of life lost. A Lancet commission,^15^ looking specifically at Africa, highlights evidence for the association between household air pollution and respiratory risks, and found that both upper and lower airway respiratory infections were associated with household air pollution. Furthermore, they found that both nasopharyngeal cancer and lung cancer were strongly associated with pollution from charcoal burning, and that chronic lung diseases, such as chronic obstructive pulmonary disease (COPD) and bronchiectasis, were associated with cooking using solid fuels. In a recent descriptive, cross‐sectional study^16^ of respiratory symptoms in 545 Ethiopian women, Tamire et al found that cough, phlegm, nose irritation, and eye irritation were significantly more prevalent among women using solid biomass fuels than among users of cleaner fuels such as liquefied petroleum gas. Using spirometry, they also found significantly lower forced expiratory volume (FEV) among solid biofuel users. Children, too, suffer respiratory effects of household air pollution from solid biomass fuels. A recent review^17^ focused on Ethiopia found that the overall pooled prevalence of acute respiratory infection in children under five, due to living in homes where cooking was done using solid biomass fuels, was 22%. Furthermore, there is a range of adverse health effects, other than respiratory, associated with solid biomass fuel exposure. A systematic review from 2020 found positive associations between wood smoke and increased blood pressure, low birth weight, esophageal cancer, sick building syndrome, non‐syndromic cleft lip and/or cleft palate, and under‐five mortality. That indoor air pollution from solid biomass fuels is a strong risk factor for hypertension was also shown by, for example, Li et al.^18^ A recent health impact assessment study^19^ estimated the burden of disease of acute lower respiratory infections, chronic obstructive pulmonary disease, ischemic heart disease, lung cancer, and stroke in an Ethiopian cohort of 2000 women and concluded that the disability‐adjusted life‐years (DALYs) lost per 100 000 women ranged between 6000 and 9000, per disease.

Additionally, household air pollution contributes to the outdoor air pollution—in rural areas it may be the major outdoor air pollution source—thereby household air pollution is responsible of an additional 400 000 deaths annually (12% of the total number of deaths attributed to outdoor air pollution). Black carbon, which is one of the main constituents of PM emitted from solid biomass fuels, have a potentially large but poorly quantified climate warming effect. As shown by Bond^20^ and highlighted by, for example, Pokhrel,^4^ Africa is the largest global source of carbon emissions from solid biomass fuel, with residential solid biomass fuels being responsible for up to 80% of Africa's black carbon emissions.

Cooking in Ethiopia is commonly conducted using charcoal in a traditional stove or wood, dung, or crop residue in a 3‐stone setup. When using a stove, the charcoal is commonly ignited outdoors to reduce exposure. A piece of plastic material, small wood sticks, cardboard, or sometimes a very small amount of gasoline or kerosene is used to ignite the charcoal, and air is supplied mechanically to assist the ignition. After ignition, the cook stove is moved indoors for cooking. In the rural parts of Ethiopia, the household energy source is mainly wood, dung, and crops residue. In these cases, 3‐stone setups are used, commonly in a separate room attached to the main room, or in the same room as family share for reading, feeding as well as sleeping. In the highland parts of Ethiopia, solid biomass fuels are used for heating in addition to cooking. Indoor sources in LICs are, hence, very different from indoor sources in high‐income countries,^21^ but still, there are a limited number of studies on solid biomass fuel burning in Africa. Most emission inventories used for Africa are based on measurements from other regions, such as Europe, North America, and Asia.^4^ Better understanding of the characteristics and exposure levels of air pollution generated by solid biomass fuels are desperately needed in order to understand the mechanisms behind the health effects, and to generate health impact assessments needed for providing policy makers with adequate input.^22^

Additionally, the Ethiopian building envelopes are generally not tight, especially in communities of low‐socioeconomic status. For example, there is often a gap between the walls and the roof allowing air to pass, and often, there can be a curtain instead of a door or of windows. This means that air exchange rates (AERs) are difficult to assess, which complicates analysis of exposure measurements, and induces uncertainties in which laboratory settings to use in order to conduct realistic experiments.

The aim of this study was to characterize airborne PM_2.5_ generated under realistic conditions in laboratory settings, to provide relevant data for indoor exposure assessments in epidemiological studies and for source apportionment studies in LICs.

## 2 METHODS

### 2.1 Solid biomass fuels

The following biomass fuels were brought from central Ethiopia: charcoal made from Eucalyptus and from Acacia, wood of Milla and of Eucalyptus, and dried cow dung. The charcoal and wood types were chosen based on them being the most frequently used in the region. Crop residue was brought from eastern Ethiopia. The fuels were purchased from street vendors or in the case of crop residue and dung, collected from the ground, and used by us as received, without modification. For charcoal, the pieces varied in size and shape, with dimensions 2–10 cm. Wood pieces were approximately 3–6 cm wide/high and 25–35 cm long, crop residue 2–4 cm in diameter and 20–30 cm long, dung was broken into pieces with approximate diameter 5 cm.

The fuels were separated into batches large enough to represent a cooking event. Each batch were weighed and kept in the laboratory (at a temperature and relative humidity of approximately 20°C and 40%, respectively) until being used.

Additionally, thin plastic bags that are often used in Ethiopia to start the charcoal fire was brought, as well as five different types of aromatic resins, in Ethiopia these are added to the charcoal directly after cooking or coffee preparation to spread their scents in the room.

### 2.2 Combustion setup

The biomass fuel aerosols were generated in a 1.33 m^3^ stainless steel chamber. Pressurized air, filtered through a HEPA‐filter, was delivered at the bottom of the volume to ensure a controlled supply of clean air for the combustion. The biomass aerosols were captured by an extractor hood with an inlet area of 0.39 m × 0.16 m placed above the fire. The hood was connected to a copper pipe, 3 cm in diameter and 1.5 m in length, through which the aerosol was transported to a larger volume by an air amplifier (Coval, M10C, Raileigh, NC, USA). At an operating pressure of 4 Bar of clean, pressurized, dry air, the air amplifier drew 400 L.min^−1^ of aerosol from the generation volume and delivered 500 L.min^−1^ into the stream of clean air generated by the air conditioning system (described below), which carried it further into the measurement volume. The extractor hood and air amplifier kept the residence time of the aerosol in the generation volume to a minimum (<2 seconds), to reduce the occurrence of aerosol dynamic effects such as coagulation, in the transportation line.

**FIGURE 1:**
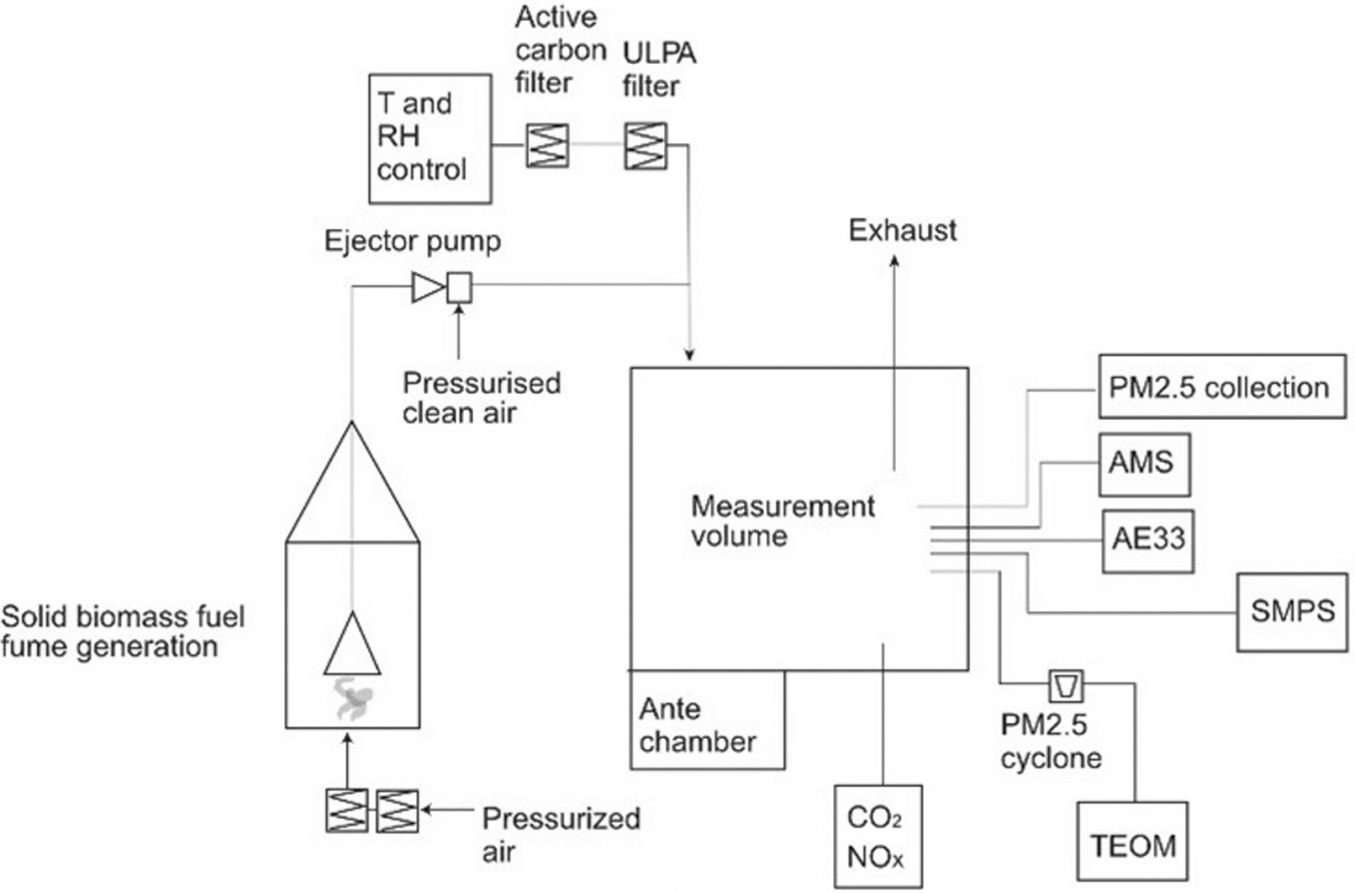
Schematic view of the complete generation/measurement system, including the measurement equipment, all highly time resolved. AE33, black/brown carbon; AMS, aerosol mass spectrometer; PM, particulate matter; PM_2.5_, particulate matter with an aerodynamic diameter < 2.5 μm; SMPS, scanning mobility particle sizer; TEOM, tapered element oscillating microbalance; ULPA, ultra low particulate air filter.

The measurement volume (used as an exposure chamber in several studies and described in detail in^23^) is a 21.6 m^3^ chamber with all interiors except for a 0.8 m^2^ window dressed in stainless steel. It can be entered via an antechamber, and the door is airtight. To avoid contamination of the surrounding laboratory, a slight under‐pressure is constantly kept in the chamber. The chamber is supplied with a controlled flow of clean HEPA‐ and active carbon‐filtered air by a custom‐built conditioning system. The flow of generated aerosol is mixed into the flow from the conditioning system just before entering the chamber at roof level. A schematic of the generation system and measurement volume is shown in Figure 1. We adapted a method to analyze PM_2.5_ particle decay rates measured with DustTrak in 26 homes in Adama, Ethiopia, after cooking (manuscript in preparation) and could thereby assess an average AER of 14.11 h^−1^ (min 3.7 and max 39.4 h^−1^). Hence, to simulate conditions in an average Ethiopian home, the AER in the chamber was set to 15 h^−1^ (the volume of the measurement volume is also in the range of typical rooms in which cooking occurs).

**FIGURE 2:**
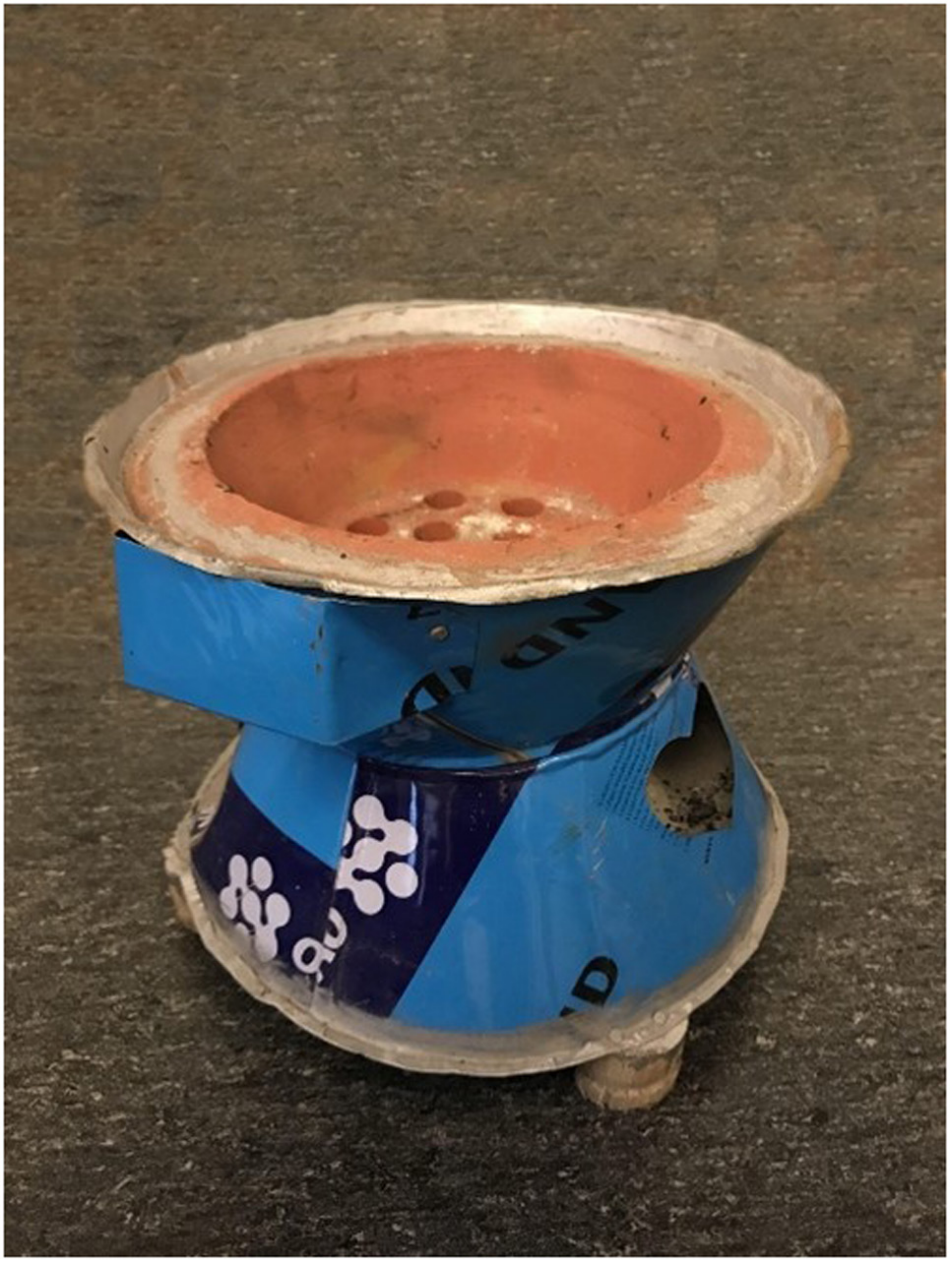
Traditional Ethiopian stove for cooking with charcoal. Approximate height and diameter: 25 cm.

The two different types of charcoal were burnt in a traditional stove (with the Amharic name Kassel Mendeja) of clay and thin metallic sheet (Figure 2) obtained at Adama market. The stove was operated by a scientist with real‐world experience of cooking with this equipment. The distance between the top of the charcoal layer and the cauldron placed on the stove (see below) was 1–3 cm. The non‐charcoal fuels: wood, dung, and crops residue were burnt using a 3‐stone setup as is commonly done in Sub‐Saharan Africa (SSA), with approximately 10 cm between fuel and cauldron. To avoid contamination from accelerants, the charcoal and dung were lit using a heating gun. The wood and crop residue were lit using matches.

For the charcoal stove, a modified water boiling test was employed. Each experiment was initiated by a “cold start” sequence in which a cold stove was fueled with approximately four hundred grams of charcoal, and lit order to bring two liters of room temperature water to boiling point. After this, a “hot start” was performed by replacing the water such that the (now hot) stove again brings two liters of room temperature water to boiling point (see Figures 5 and 7). This second batch of water was left on the stove simmering until the charcoal was consumed. The main deviation from the standard water boiling test^24^ was that we used a lid (as cauldron users are prone to do in everyday use) to reduce heat lost from the water. For the non‐charcoal fuels, our full modified water boiling test was found unfeasible, but the cauldron and the 2 liters of water were kept, as quenching of flames likely influences emissions. Non‐charcoal fuels were thus burned in a similar manner as the charcoal, the difference being we did not achieve full test cycles (cold start and hot start described above).

In selected experiments, we also explored the effects of plastic bags (procured at the Adama market) used as accelerants to light the stove, as this practice is common and likely to result in emissions. Furthermore, we also investigated particle emissions from aromatic resins (also from the Adama market), which are added by some stove users to the charcoal post‐cooking/coffee ceremony to produce fragrance. These emissions are not included in the modified water boiling tests reported, for those we aimed to characterize fuel emissions only, for clarity. We include the bag and resin emissions as examples of non‐fuel emissions from stove operation.

## 3 INSTRUMENTATION

### 3.1 On‐line measurements

Mass concentration of PM_2.5_ was measured with a Tapered Element Oscillating Microbalance (TEOM, Rupprecht & Patashnic Co Inc., Albany, NY, USA) at 50°C with a cyclone (BGI Inc., USA) as pre‐collector for particles larger than 2.5 μm at 4 L.min^−1^ (achieved by splitting the 4 L.min^−1^ flow after the pre‐collector so that 1 L.min^−1^ was drawn by the TEOM and 3 L.min^−1^ by the vacuum system). In a selection of the experiments, a portable DustTrak (Model DRX 8533, TSI, Shoreview, MN, USA) were used in addition to the TEOM, to study its feasibility in monitoring solid biomass fuel emissions. The DustTrak was zero‐calibrated prior to each experiment using a HEPA filter according to the standard procedure recommended by the manufacturer.

Mobility size distributions and number concentrations of particles 10–500 nm were measured by a Scanning Mobility Particle Sizer (SMPS, Model 3082, TSI, Shoreview, MN, USA), consisting of a 44 cm differential mobility analyzer (DMA, Model 3082) and a condensation particle counter (CPC, Model 3772). The aerosol flow rate was 0.35 L.min^−1^, and the sheet flow was 1.05 L.min^−1^. An aerosol particle sizer (APS model 3321, TSI Inc., USA) was used in early experiments to verify that there were very low concentrations above 500 nm (data not shown) in our setup.

An aerosol mass spectrometer (AMS, Aerodyne Inc., Billerica, MA, USA) was used for online chemical characterization of the emitted PM. The ionization efficiency (sensitivity) of the instrument was calibrated using 300 nm ammonium nitrate particles, size selected with a differential mobility analyzer (DMA Model 3082) and counted by a condensation particle counter (CPC, Model 3772). A soot particle AMS was used, but here we report data recorded with the “soot module” (laser vaporizer) disengaged, hence only “non‐refractory,” which flash vaporizes at 600°C PM is included in the mass spectra. The vapors produced from the particles are ionized with 70 eV electrons, followed by time‐of‐flight mass spectrometry. The spectra were analyzed with SQUIRREL 1.63I and PIKA 1.23I.

Light extinguishing aerosols such as black carbon were measured by an aethalometer (AE33, Magee Scientific, Berkeley, CA, USA). The AE33 measures at 7 optical wavelengths, from 370 nm to 950 nm, giving it the ability to specifically detect the more brown (rather than black) PM, which is often used as an indicator of biomass combustion in atmospheric data.

NO_x_ was measured with a chemiluminescence analyzer (CLD 700 AL, ECO PHYSICS AG, Switzerland).

PM_2.5_ emission factors were calculated for charcoal, wood, and dung based on carbon balance, that is, by tracking the carbon in the fuel.^25^ CO_2_ from the biofuel combustion was detected with a LICOR analyzer (LI‐8020 Li‐COR, Lincoln, NB, USA). Background CO_2_ levels of around 400 ppm, corresponding to about half of the measured CO_2_, were subtracted, and the remaining CO_2_ was attributed to biofuel combustion. For the emission factor calculations, we approximated the following carbon fractions in the fuel; charcoal: 1, wood: 0.5, and cow dung: 0.5.

### 3.2 Off‐line measurements

PM_2.5_ cyclones (BGI Inc., USA) were used to collect particles on 37 mm quartz filters for organic carbon and elemental carbon (OCEC) analysis and on 37 mm cellulose esters filters (Millipore, pore size 0.8 μm) for elemental analysis with Particle Induced X‐ray Emission (PIXE). Both filter types were mounted in conductive three‐piece filter cassettes (SureSeal, SKC Inc., USA), and sampler pumps (BGI 400, BGI Inc., Waltham, MA, USA) at 4 L.min^−1^ were used for the collection. The planned carbon analysis could not be conducted due to that the thermal‐optical carbon analysis system was offline. In the PIXE analysis, conducted at the Division of Nuclear Physics at Lund University, a 2.55 MeV proton beam is used to generate element‐specific X‐ray emission lines.^26^ In these experiments, PIXE was used to compare the relative abundance of different species from different biomass fuels.

Additionally, a high‐volume sampler (BGI 900 BGI Inc., Waltham, MA, USA) was used at 900 L.min^−1^, to collect PM_2.5_ from the different biomass fuels for later toxicological in‐vitro studies (not presented here).

## 4 RESULTS

### 4.1 Emission factors and exposure levels

**FIGURE 3:**
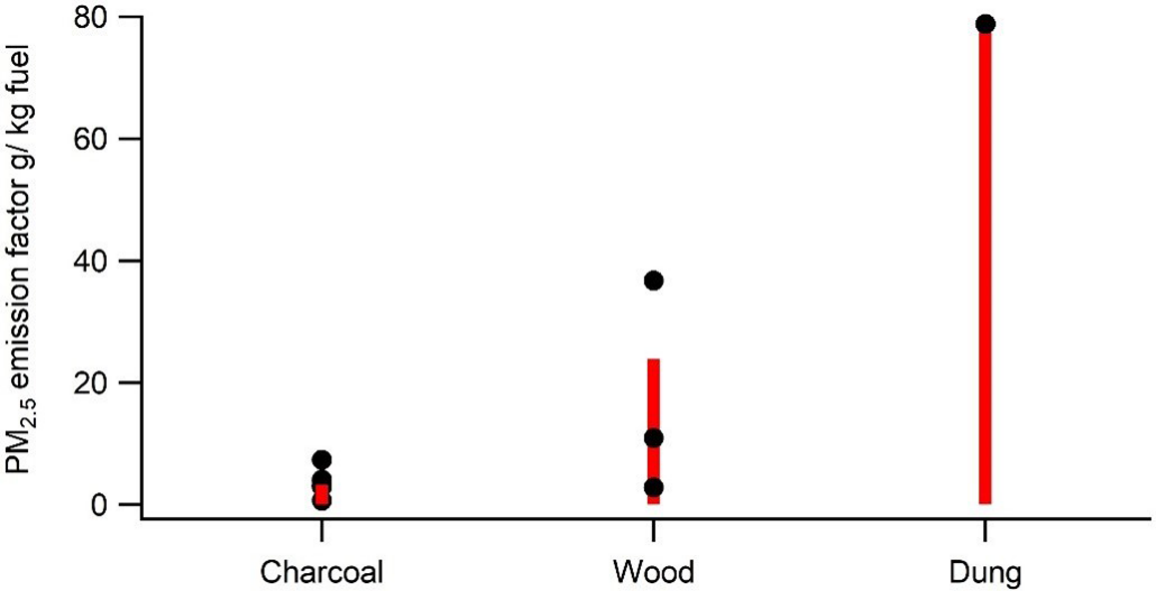
Emission factors for charcoal (Eucalyptus and Acacia), wood (Milla and Eucalyptus), and dung, based on TEOM data. The black dots are results from individual experiments (*n* = 7 for charcoal and *n* = 3 for wood), and height of the red bar shows the average for the fuel.

Emission factors (in units of g PM_2.5_/kg fuel) were calculated based on TEOM (see Figure 3) and DustTrak data. The DustTrak gave similar emission factor results for charcoal (the DustTrak to TEOM ratio was 0.8–1.1) but yielded threefold higher emission factor values for wood and dung, with a DustTrak to TEOM ratio of 2.8 for both biomass fuels, highlighting the need to validate DustTrak measurements for each type of aerosol investigated.

From the TEOM data, average PM_2.5_ levels in the measurement volume (the simulated cooking environment) were calculated during a full burning event (from that the concentration started to increase to when the concentration had declined back to baseline) for the different fuels. The results are shown in Table 1.

**TABLE 1:** Examples of PM_2.5_ levels in the measurement volume which represents the residence in which cooking occurs, calculated from individual burns for each fuel

Solid biomass fuel type	Average PM2.5 concentrations (μg/m^3^) at AER 15 h^−1^	Peak PM2.5 concentration (μg/m^3^) at AER 15 h^−1^	Average PM2.5 concentrations (μg/m^3^) per 100 g fuel at AER 15 h^−1^
Charcoal Acacia	320	700	630
Charcoal Eucalyptus	310	700	170
Wood Milla	8300	25 000	8000
Wood Eucalyptus	6700	20 000	3600
Crop residue	210	420	200
Dung	16 000	49 000	22 000

The PM_2.5_ mass concentration levels generated from wood are more than one order of magnitude higher than from charcoal, and the levels generated by dung burning are yet another order of magnitude higher. The crop residue PM_2.5_ levels were surprisingly low, and more experiments are needed to confirm whether those levels are representative. See Figure 3 for an overview of repeatability of charcoal (*n* = 7) and wood (*n* = 3) results.

### 4.2 Size distributions

**FIGURE 4:**
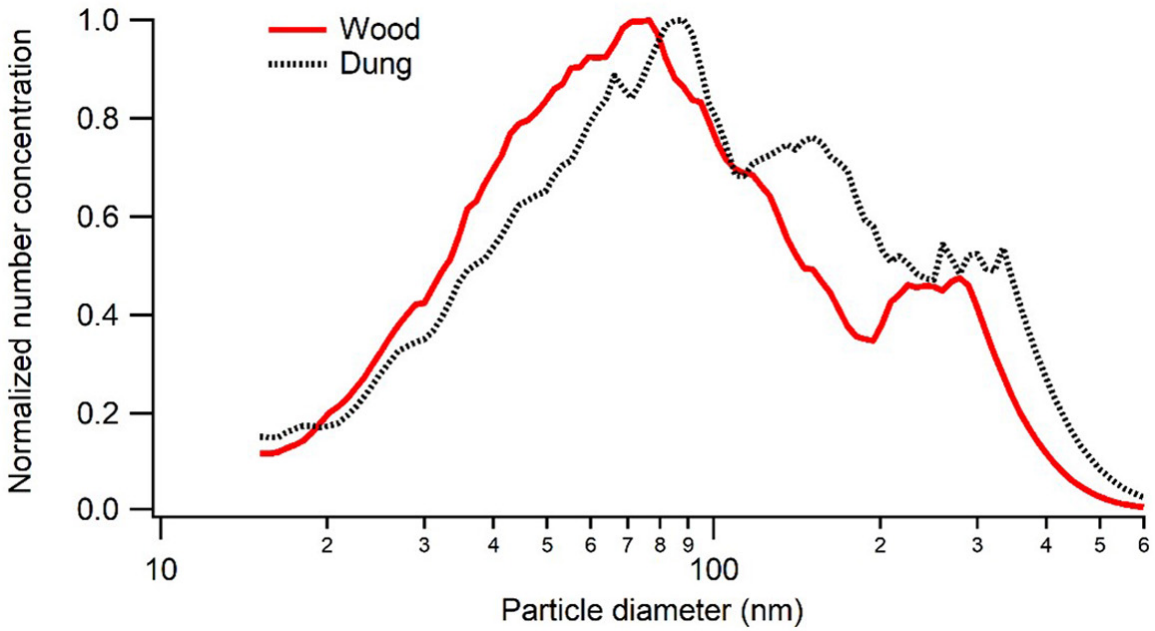
Normalized size distributions from wood combustion and dung combustion measured by Scanning Mobility Particle Sizer (SMPS). The data are normalized to account for “bin width” and rescaled such that the highest value is 1.

We found, consistent with many previous studies, that solid biomass fuels predominantly generate airborne particles of small sizes. Figure 4 shows an example of size distributions for particles from wood and dung combustion. Both size distributions are clearly multimodal, and the dominating mode, in terms of particle number, is below 100 nm.

Other experiments (not shown) in which transport from generation volume into measurement volume (i.e., the simulated residence air) was less rapid (see “combustion set‐up” in the method section) produced monomodal distributions with larger particles, though never larger than a few hundred nanometers in diameter. This reflects the fact that coagulation between particles tends to produce larger sizes as time passes by. For example, downwind of the cooking, or after some time when lower PM levels remain in the residence. As the size distributions from different fuels were not that different to begin with, and end up even more similar (and unimodal), particle size is clearly a poor marker for their origin.

### 4.3 Chemical composition

**FIGURE 5:**
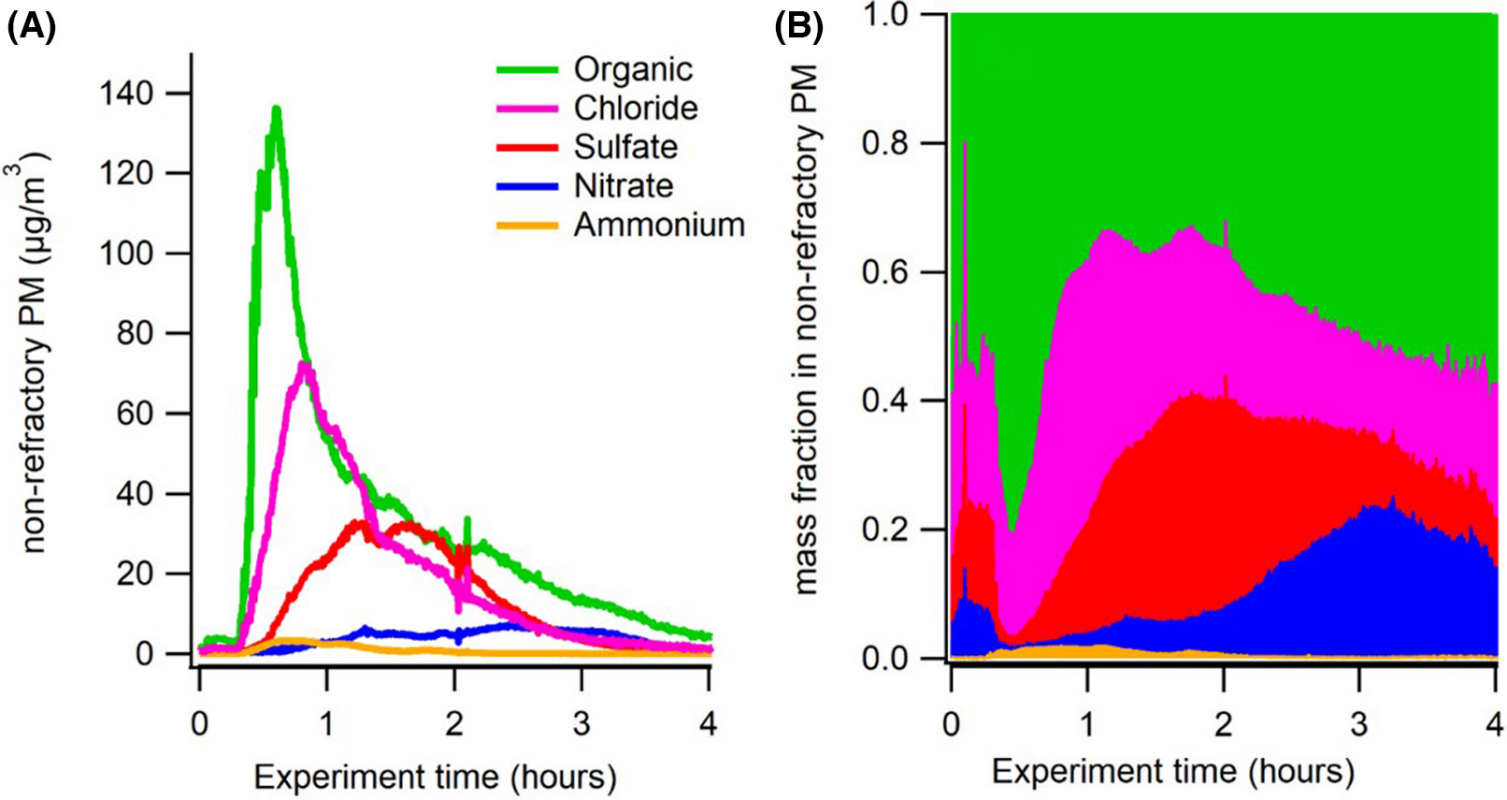
Time resolved chemical composition of PM from Eucalyptus charcoal burning measured by the AMS. Chemical species concentration (A) and fraction (B). Non‐refractory refers to readily vaporized (at 600°C).

The chemical composition of the PM_2.5_ emitted from charcoal combustion varies with time after ignition. Figure 5 shows an example of charcoal combustion, where it can be seen that there is an initial high peak in the PM_2.5_ mass concentration, with a high fraction of organic compounds. This is true also for the other fuels (not shown here). This peak is followed by a build‐up of inorganic PM. Chloride level is the next to rise, peaking around half the peak organic concentration. Sulfate rises next, to approximately half of peak chloride concentration. This is followed by a smaller amount of nitrates which builds up over longer time.

**FIGURE 6:**
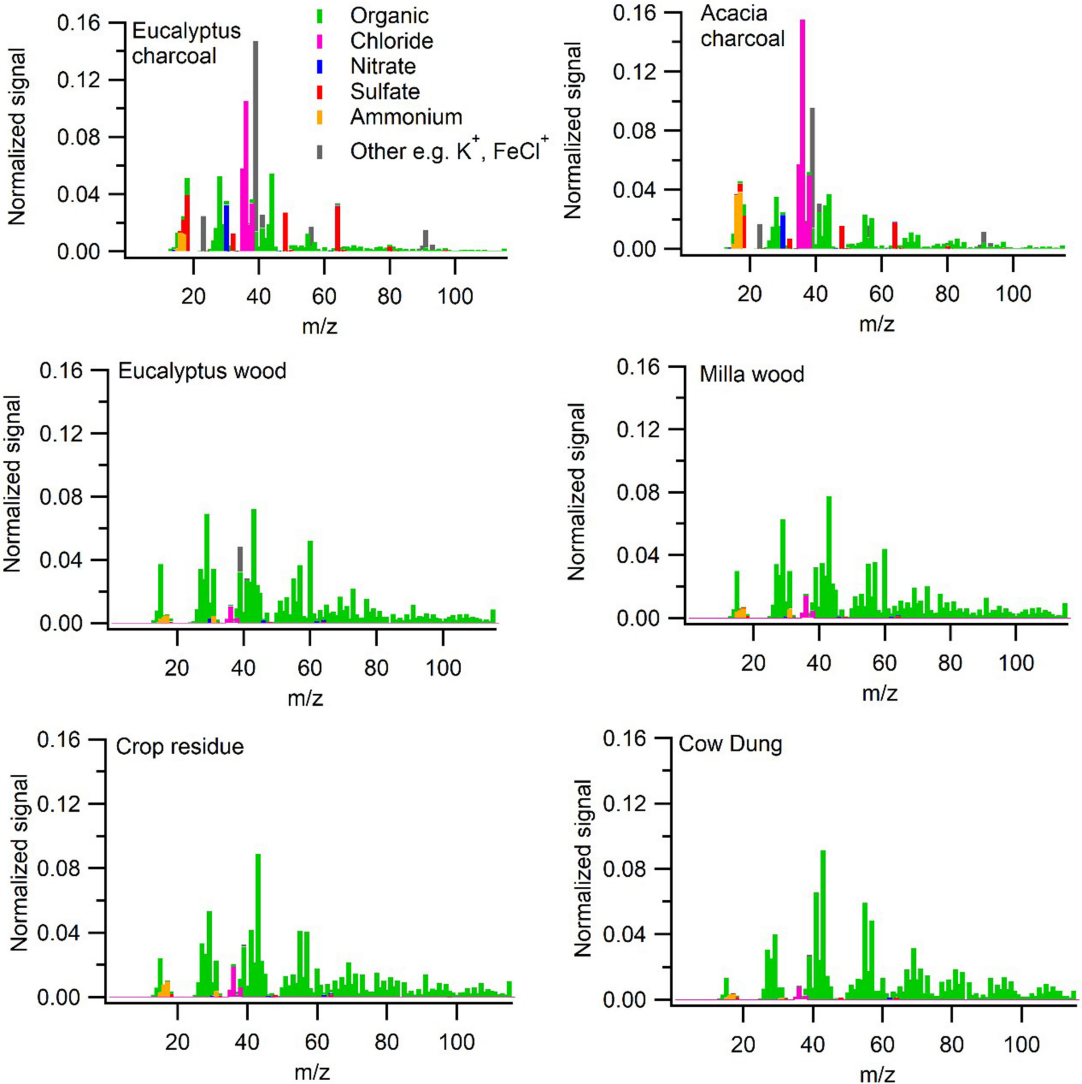
PM mass spectra from the investigated fuels, normalized for comparison (such that the sum is 1 for each spectrum, an at each mass to charge ratio, *m/z*, the signal fraction can be read.) Each spectrum is the average from one burning session, representative for the sessions with this fuel.

Charcoal is different from the other fuels in that the non‐refractory PM emitted contains comparable amounts of organic and inorganic material, while the others are dominated by organics, as can be seen in Figure 6. Eucalyptus and Acacia charcoal combustion produced rather similar mass spectra. The main peaks are HCl^+^ (at *m/z* 36) and K^+^ (at *m/z* 39), with Eucalyptus yielding higher K^+^ than HCl^+^, and the opposite (HCl^+^ > K^+^) for Acacia.

Notably, the charcoal spectra contain low abundances of the source‐tracer‐relevant levoglucosan fragments C_2_H_4_O_2_^+^ and C_3_H_5_O_2_^+^ (at *m/z* 60 and *m/z* 73, respectively), while these are abundant in the wood‐burning emissions, both Eucalyptus and Milla (this is true also when comparing the organic signals only). The non‐charcoal burning emissions are different in that a large fraction of the signal (22%–29%) is due large ions (in an AMS context) at *m/z* > 115 (not shown). Burning crop residue and dung produces PM which gives rise to less of the levoglucosan marker peaks compared to wood‐burning emissions (yet more than that of charcoal) as recently reported for dung combustion by Loebel Roson et al.^26^ In fact, *m/z* 60 in the dung spectrum, while usually (for non‐dung‐burning emissions and most ambient aerosol) dominated by C_2_H_4_O_2_^+^, here has a strong contribution also from CH_2_NO_2_^+^ (ca 40%). Overall nitrogen atoms account for ~7% of the signal in the dung‐burning mass spectrum, which is unusually high for organic PM (as it is normally dominated by carbon, oxygen, and hydrogen). Crop residue emissions have about half the nitrogen contribution dung has, and in the wood and charcoal burning spectra, nitrogen is lower still.

**FIGURE 7:**
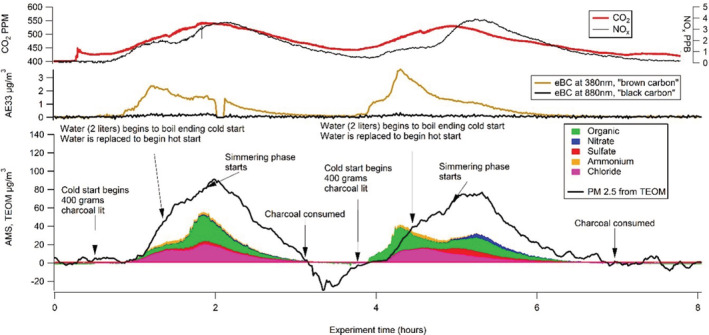
Modified water boiling test using charcoal. Gas concentrations on top panel, “equivalent Black/Brown carbon” on middle panel (equivalent in the sense that although we measured light extinction, we report estimated mass concentration, as is commonly done) and PM_2.5_ as well as “non‐refractory” components therein. Details in main text.

From the PIXE analysis, it could be seen that all fuel emissions were dominated by potassium, chloride, phosphorous, and silica. The two types of charcoal, as well as Milla wood, were more dominated by potassium than the others. It should be noted that PIXE is “blind” to the organic components shown in the AMS results (Figures 5, 6, 7), which are very abundant in the non‐charcoal emissions. The Eucalyptus charcoal and the Eucalyptus wood emissions have a higher relative content of chloride than the other fuel emissions as measured by PIXE.

A typical modified water boiling test with charcoal is illustrated in Figure 7. As can been seen, there is a discrepancy between AMS and TEOM concentrations. The fact that TEOM typically shows higher concentrations is likely due to the presence of “non‐refractory” (resistant to vaporization) salts which are not detected by the AMS (in this configuration), that is, the AMS is “missing” much of PM_2.5_ in charcoal burning emissions. The TEOM, on the contrary, shows negative (unphysical) values for the end of the first charcoal batch. This is likely due to evaporation of volatiles from the TEOM filter. As the more volatile species are “non‐refractory” that is, measurable with AMS, it is likely the artifacts in each instrument arise from different PM components. The aethalometer (AE33, middle panel) shows very low concentrations of light‐absorbing aerosols (considering PM concentrations), which are brown rather than black. NO_x_ levels are low compared to PM levels throughout the experiment.

**FIGURE 8:**
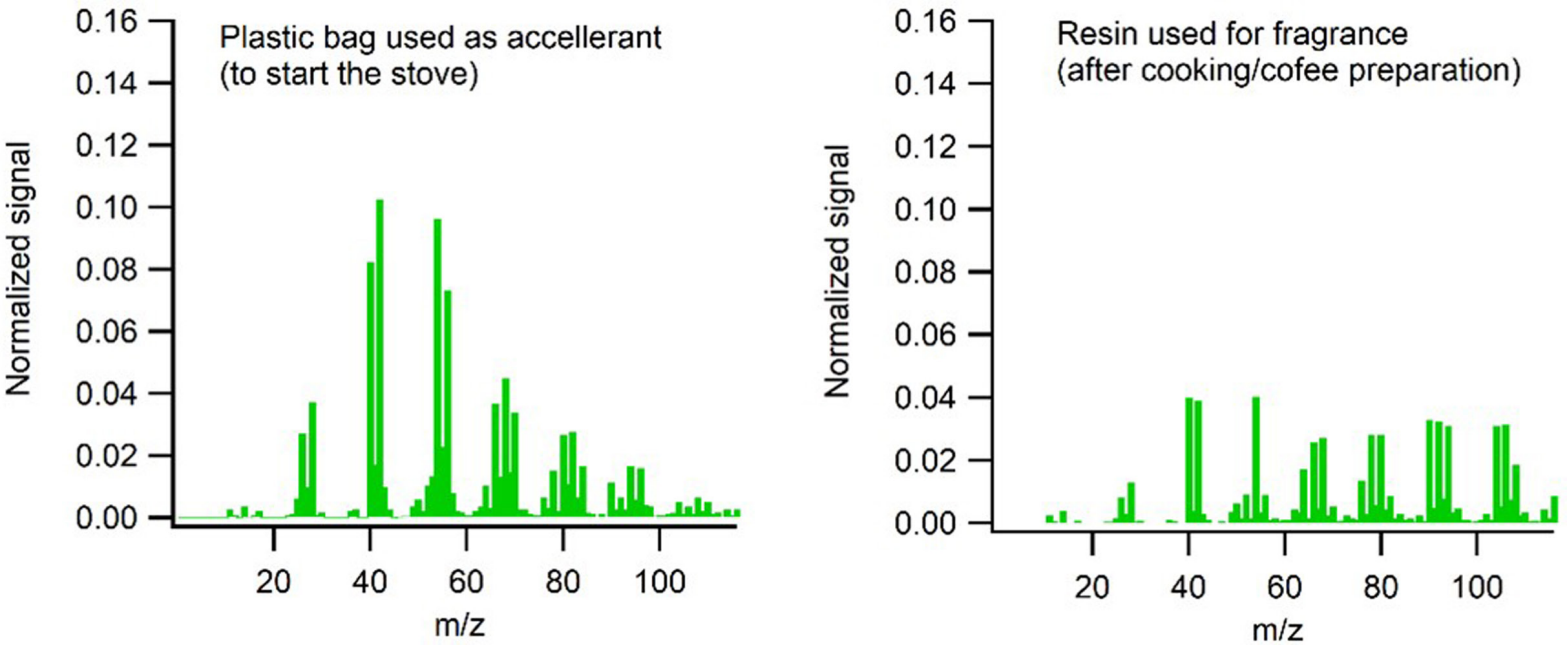
PM mass spectra from non‐fuel combustion emissions related to stove operation, resin and plastic bag. Normalized for comparison (such that the sum is 1 for each spectrum, and at each mass to charge ratio (*m/z)* the signal fraction can be read.)

Figure 8 shows mass spectra (heavily dominated by organics) for plastic bag and aromatic resin combustion. Both are distinctly different from the solid fuel combustion mass spectra shown in Figure 7. However, the plastic bag burning emissions have similarities both to fresh traffic exhaust and cooking organic aerosol as observed in, for example, European datasets, which could confound future source attribution efforts in Sub‐Saharan Africa (SSA). We investigated 5 different resins procured at Adama market and found very similar mass spectral signatures for them. Note that the low (normalized) signal intensities shown for resin do *not* indicate low emissions (we do not quantify the amount of non‐fuel emissions, for example, in terms of emission factors as done for the fuels, in this work). It reflects the fact their mass spectra are characterized by very high (~50%) signal fractions above *m/z* 115, indicative of thermally stable molecules.

## 5 DISCUSSION

In this study, we have looked at physicochemical properties of biofuel PM_2.5_ relevant for exposure assessment and epidemiological studies. The PM_2.5_ was generated in a laboratory setup under realistic conditions, that is, using a combustion initiation and procedure that is used in real life and studying the emissions at a real‐world‐relevant air exchange rate. Concerning both exposure levels and chemical composition, there is a difference between the different fuels (charcoal, wood, crops residue, and dung). Titcombe et al^27^ measured indoor PM_2.5_ in a pilot study in homes in Tanzania and found average exposures during cooking events of 1574 ± 287 μg/m^3^ when using firewood and 588 ± 347 μg/m^3^ when using charcoal, which is in the same range as in our laboratory experiments. In a study of 150 households in rural Ethiopia, Tamire et al^28^ found that the average duration of cooking per 24 hours was 258 minutes. Assuming that cooking (including coffee making) is conducted for a total average of 4 hours per day, the 24‐hour average exposure due to cooking alone would be 53 μg/m^3^ using charcoal (average of the two types in this study), 1300 μg/m^3^ using wood (average of the two types in this study), 35 μg/m^3^ using crops residue(a remarkably low value in this context which motivates further experiments for verification), and 2700 μg/m^3^ using dung. Even for the lowest emitter in this study, crops residue, the exposure levels at an AER of 15 h^−1^ exceed double the WHO air quality guideline^7^ of 15 g/m^3^ as a 24‐h average of PM_2.5_, and for dung, the exposure level was well over 100 times higher than the WHO air quality guideline. It should, of course, also be noted that the exposure that can be derived from this study only includes emissions from the solid biomass fuels themselves, not the additional exposure that is generated by the food that is being cooked. To assess indoor exposure in epidemiological studies have long been a challenge. Several studies of emission factors have been done, but to use these emission factors, information on mass of fuel being used is needed. This information is seldom available since it is not feasible to ask all households that are part of a large cohort to weigh their fuel before igniting it, and to weigh the residues of the fuel after cooking. Information that is easily available is type of fuel used and time per day spent on cooking. We suggest that using an approach that assesses PM_2.5_ concentrations in a controlled laboratory volume with a relevant air exchange rate, in our case 15 h^−1^, might be a more feasible way to assess indoor exposure in large LIC cohorts. Further work is recommended comparing ways of assessing indoor exposure by combining field and laboratory experiments to find the best strategy to advance epidemiology.

From the time‐resolved measurements (see Figure 5), it could clearly be seen that the peak exposures occur very early in the combustion process. The AMS measurements revealed that this is due to that volatile organic compounds, are released from the fuel in the heating process. A large amount of the exposure caused by this initial peak is avoided if the charcoal is ignited outdoors, as often is customary, and the stove carried indoors first when the charcoal is properly lit. We observed discrepancies between the PM concentrations as measured/estimated from TEOM, AMS, and dustrack. This highlights the need to use complementary particle measurements tor evaluation of data products through “closure” of, for example, PM_2.5_ (making sure they are in fair agreement and quantifying their discrepancies). This should be seen as a long‐term goal for ambient monitoring/field campaigns in SSA and an important aspect to consider in laboratory investigations of SSA‐relevant sources.

As expected, the absolute majority of the particles generated in the study were smaller than 2.5 μm. In fact, an aerosol particle sizer (APS) was initially installed to measure number concentration and size distribution of particles 0.5–20 μm, but since almost no such particles were detected, the APS was not used for the rest of the experiments. Other studies, such as,^12^ with solid biomass fuels conducted in laboratory settings have come to the same conclusions regarding particle sizes.

The choice of AER in laboratory studies plays a big role in what aerosol dynamics that can be expected. When the air exchange is low there is more time for coagulation, facilitated by the high particle number concentrations generated by combustion, leading to a shift with time toward larger sizes in the particle size distribution. Even at the AER used in this study, 15 h^−1^, which is relatively high compared to the average AERs of 0.5–1 h^−1^ in high‐income countries,^29^ we could see a shift in particle size, during the course of 45–60 minutes the average mobility diameter was doubled. Particle size is of importance since it governs where in the lung the particles are likely to be deposited upon inhalation. Particles with a diameter of 50–100 nm are most likely to reach the alveoli and have a deposition probability there of up to 50%. Several different factors affect if the particles can initiate adverse effects once deposited, one of these being the chemical content of the particles. As can be seen in the chemical analysis by AMS and PIXE, the chemical composition of the PM varies with different solid biomass fuels. These differences in chemical composition, which should ideally be confirmed using targeted field measurements, indicate that the health effects of the PM are likely to be different from different fuels, even at the same concentrations, a fact that could be seen in the toxicological pilot study that was conducted with the particles collected by the high‐volume sampler (conference abstract,^30^ manuscript in preparation). It was found that particles derived from all three types of solid biomass fuels were cytotoxic to bronchial epithelial cells, but that wood and dung inducing the highest toxicity. Elucidating the toxicological mechanisms responsible for damages from solid biofuel combustion is a complex task, nitrogen‐containing organic compounds^26^ and metals^31^ have been suggested to play a role in dung‐burning PM toxicity. NO_x_, which is known to cause both cardiovascular effects^32^ and serious adverse birth outcomes,^33^, ^34^, ^35^, ^36^, ^37^, ^38^ was emitted in relatively low amounts in our experiments. The high NO_x_ levels of 86.5 μg/m^3^ that have been reported outdoors in, for example, Adama, Ethiopia,^39^ are, hence, likely not caused by the extensive charcoal burning typical of that area.

Chemical analyses are also of importance for source apportionment. For high‐income countries, levoglucosan, which forms from thermal processing of cellulose, is often used as a tracer for wood burning. Judging from the low amounts of levoglucosan fragments detected by AMS in charcoal PM, cellulose does not “survive” the charcoal production (and hence, levoglucosan is not emitted from charcoal combustion). (Alternatively, the charcoal combustion conditions do not favor levoglucosan formation from the cellulose remaining in the fuel. The implications for source apportionment are the same in this scenario.) Dung‐burning emissions were found to yield some levoglucosan fragments (though much lower than wood), and it seems likely the diet of the animals plays a role in governing this. Thus, levoglucosan should be interpreted with caution, and ideally informed by knowledge of the fuel mixed used upwind of the field measurement site, in LIC contexts.

Although our experiments show comparable levels of chloride and organic PM from charcoal combustion, it is likely atmospheric processing increases the organic fraction after emission through formation of secondary organic aerosol (gas phase emissions which age chemically into products which partitions into PM). However, as each household is primarily exposed to their own stove emissions, chloride is likely prevalent in the inhaled PM.

Optical properties of PM are also used to apportion sources. Our data suggest low brown carbon and even lower black carbon from charcoal while wood combustion produced more absorbing emissions. “Brownness” as parametrized by Aangstrom absorption exponent is often used to estimate solid fuel emissions and distinguish them from traffic sources, this should be explored taking into account LICs have different black and brown carbon sources than high‐income countries. This study could (combined with others) be used to guide coming field measurements in LICs from which source apportionment can be drawn. A correct source apportionment is of high importance for verifying exposure assessments and important input for interventions and policy making.

To assess the full exposure situation, and its potential health effects, close collaborations between epidemiology, aerosol science, and toxicology are crucial. Outdoor exposure is only one part of the picture, and indoor exposure assessments, especially in LICs, are challenging. Experiments, such as,^4^ conducted to fit standardized test protocols are of importance when it comes to understanding the emissions, but in order to relate the emissions to real‐world exposure, experiments that mimic real‐world cooking scenarios are equally important. Physicochemical characterization performed under real‐world like conditions and PM collection for toxicological studies can, combined, provide crucial guidance to epidemiological studies. This type of interaction can help cross‐fertilize all three fields of research and, foremost, generate currently lacking important data on exposure in LICs.

The lack of detailed exposure data is currently creating a negative feedback loop, which cross‐disciplinary collaborations can help stop. As long as there is a lack of reliable data on exposure and of the health consequences of this exposure, public awareness and public concern will be missing. Without public concern, there will be no policy actions. If there are no policies that needs to be followed, there will be very little incentives to monitor the air and collect data, even though the costs in both health care and diminished economic productivity due to air pollution currently correspond to billions of USD^15^ in several Sub‐Saharan African countries. Apart from the economic costs, the human suffering from air pollution‐related deaths and diseases is massive, and thus, science‐based mitigation actions are clearly needed.

The severe lack of air pollution data from the SSA region is an obstacle for mitigation. We suggest that this is addressed by first focusing on the emission sources that are most common in the region. For indoor air pollution, this is biomass burning. When we can make exposure assessments where this source is included, we should move forward and address whether there are significant differences within the region, and also between the region and other regions, when it comes to, for example, differences in composition between fuels, user behavior, or combustion appliance, and the resulting emissions.

## 6 CONCLUSIONS

All solid biomass fuels generated high levels of PM_2.5_. Concerning exposure, there is a big difference between the different fuels, with the less energy dense fuels, such as dung, generating the highest amount of PM_2.5_. The majority of PM from all the biomass fuels were smaller than 300 nm, a particle size fraction which has high deposition probability in the alveoli tract of the lung. It is high time to import state‐of‐the‐art approaches from aerosol science, such as in‐depth physicochemical characterization of different sources, to provide a more detailed exposure assessment beyond only mass concentration of PM_2.5_. Better understanding of real‐world emissions and exposures are also much needed input to toxicological studies. Additionally, studies like this one helps us gain a better understanding of the links between activities (such as cooking and heating, transport, industry) and air pollution, which is much needed for policy making, not least in LMICs. In order to assess indoor exposure in detail using real‐world like laboratory settings, as well as assess the health outcomes of that exposure and the underlying mechanisms, a close collaboration between aerosol science, epidemiology, and toxicology is necessary.

## CONFLICT OF INTEREST

We declare no conflict of interest.

## Data Availability

The data that support the findings of this study are available from the corresponding author upon reasonable request.
